# The President’s Address1Read at the Thirty-eighth Annual Meeting of the American Veterinary Medical Association, Hotel Rudolf, Atlantic City, N. J., September, 1901.

**Published:** 1901-11

**Authors:** Tait Butler

**Affiliations:** Raleigh, N. C.


### THE PRESIDENT'S ADDRESS.[1]

By Tait Butler,

RALEIGH, N. C.

(Concluded from page 635.)

This Association has a written Code of Ethics, an observance of which is required from every member. The restraining influence thus obtained has had a splendid effect in elevating the standard of veterinary ethics, not only among our members, but also throughout the whole profession. It is true that in the light of modern ideas concerning the rights of the individual and more liberal views of business enterprise some sections of our code may seem somewhat antiquated and contracted, but it has served a good purpose, and this Association should now require the same high standard of moral and business integrity which it has enforced in matters strictly professional. It is needful that we look more

[1] Read at the Thirty-eighth Annual Meeting of the American Veterinary Medical Association, Hotel Rudolf, Atlantic City, N. J., September, 1901.

carefully into the moral character and business standing of our applicants for membership. The strength of our organization is as much dependent upon the high moral and business integrity of our members as upon their professional and scientific attainments.

I have thus spoken of veterinary ethics in a general way, but I wish to particularly call your attention to a violation of our code, in spirit at least, by some of our veterinary colleges. This is a matter that can only be corrected by the action of this Association, and I recommend that it receive careful consideration at this meeting. I refer to the practice of certain veterinary colleges in illustrating their catalogues and advertising publications in a manner that would not be tolerated for an instant if done by the individual member. The college which uses illustrations in its advertising literature for any other purpose than to show the extent and variety of its buildings and equipment has descended to the level of the practitioner who uses illuminated stationary and flaming posters. In fact, such exhibitions of bad taste are much worse on the part of the college than in the individual, for the college should be the model for its graduates. What alma mater does the alumni are not likely to think wholly bad; therefore, it would not be strange if the graduates of such schools held rather cheaply that section of our code which forbids such practice. I have here a few samples of this sort of cheap, undignified, and unprofessional advertising to which I invite your attention.

We have all heard with regret of the failure of the profession in its efforts to secure proper recognition for the army veterinarian, and to give to the army a veterinary service commensurate with the importance of the interests involved. But this partial failure must not cause us to relax our efforts. Your committee, although in a measure unsuccessful, has done valiant service and manufactured much public sentiment in behalf of our cause, for which it deserves the sincere thanks of the Association, and I feel certain we shall all be interested in any plans for future action which it may be pleased to suggest.

There are other fields offering more fruitful harvests which should no longer be neglected. This Association should appoint a committee and through it inaugurate a campaign which should be prosecuted with the utmost vigor, and only cease when every board of health and every sanitary commission in the land has at least one competent veterinarian in its membership. I would most respectfully urge the appointment of such a committee.

Each succeeding year marks a distinct advance in the magnitude of the Association's influence and usefulness. Our field of labor is becoming more comprehensive, comprising more diverse and special interests. From sessions lasting one day we have reached the limit in this meeting of a programme covering four full days. Four days, with the time required to go to and from the meeting, consumes the entire week, and one week is all the busy veterinarian is willing to give to such a meeting. We have, therefore, reached the limit of the time at our disposal, but have we reached the limit of the requirements? The history of the last three years makes it appear quite certain that we have not.

This is the age of specialization, and veterinary science offers no exception to the rule. No man is any longer able to cover the whole field of veterinary work. It matters not whether we recognize and admit it or not, the fact remains that the time has come when the veterinarian must begin to specialize if he is to attain prominence. What applies to the veterinarian also applies to this Association. We have accomplished the basic work which must always precede successful specialization, and the time is near at hand when we must be prepared to so change the machinery of the organization as to meet the requirements of modern ideas and methods. In fact, in my opinion the time has already arrived when there is a real necessity for a reorganization of our plans of work to meet the needs of the diversity of interests involved. The field being too broad for the grasp of any one mind and too long for the time at our disposal, we are forced into the organization of special sections for special lines of work. We have for some time been trying to shut our eyes against the fact, but even the blind must see that "section work" has been going on for some time. Last year we had clinics absorbing greater interest from the general practitioner than the scientific literary programme. We also have our companion organization of experiment station veterinarians. The natural divisions which have spontaneously shown themselves give us a clue to the sort of reorganization demanded. Accordingly, it appears to me that three sections working concurrently—one considering questions of interest to the general practitioner and including the clinics; one dealing with sanitary questions, including meat- and milk-inspection and general State medicine, and one comprising investigators, experiment station workers, etc.—would meet present needs. I suggest, therefore, and recommend that a committee be appointed for the purpose of considering the advisability of "section work" and formulating a plan for putting the same into operation, if deemed advisable, and report the same to the next annual meeting of the Association.

For several years I have thought the necessity for at least two of our standing committees, as their duties are now defined, was not very apparent. I refer especially to the Committee on Intelligence and Education and the one on Diseases. The utility of the former is not obvious, while the latter must have its duties better defined and its efforts concentrated before it can accomplish material good. During my term of office these facts have been brought to my attention quite forcibly by similar ideas suggested by various members. I believe certain members of these committees will have something to say along this line, and I need not discuss the matter further in this connection.

No feature of these annual meetings is more attractive nor more beneficial than the opportunity which they offer for the enjoyment of social intercourse between congenial spirits. The chance acquaintance of long ago has each year grown into a closer and warmer friendship, until at last on the day preceding the meeting we watch and long for the old familiar face which we have learned to love in the years gone by. If perchance the expected one fails to appear and we know that he is only temporarily detained, but will join us at the next and perhaps many subsequent meetings, the disappointment may be sharp, but the hearty hand-clasp and warm greeting of another dissipates the sadness and cheers the heart as only the feelings of true friendship can cheer it. But when cruel and relentless death has thrown its dark pall between us and some dear friend, and we gather to miss his genial presence and are reminded that the chief among our friends will never again join us in these councils, where his wisdom is so much needed, that we shall never again enjoy that companionship which was so much prized, yea, that the brother we loved is no more forever, we are truly sad, and the heartache cannot be soothed. There is a void which can never be filled. Other friendships, perhaps just as warm and true, may and will be made, but the place left vacant forever remains the same.

During the past year several worthy members of this Association have been lost to us through the grim harvester Death Among these were two who had been especially honored by election to the presidency. The one, ripe in years largely devoted to the advancement of the profession, few of you here assembled knew personally; but the other, in the fulness of his vigorous manhood and the geniality of his noble presence, was known to all. And in this case it may be truly said that to know was to love. I refer to our late friend and brother, Dr. Albert W. Clement. I cannot allow this fitting opportunity to pass without paying my humble tribute to his memory. I shall always fondly cherish the memory of a friendship which was one of the first made among the members of this Association. No greeting was more cordial, no hand-clasp more hearty, no friendship more true than that which he gave and won in return from all who came to know him well. His scientific mind, his conscientious investigations, and his conservative yet fearless exposition of all that was best in progressive modern veterinary science won the highest respect from us all. But it was his genial personality which made him a companion to be enjoyed and loved, and his broad nobility of character that won the affection and held the hearts which now mourn his loss.

---

TETANIC INFECTION THROUGH THE DIGESTIVE TRACT. Military veterinarian Rémond treated a horse in the infirmary from November 8th to 13th for congestive colic. On the 16th following the horse was returned with a history of abdominal pain, but an examination revealed all the classical symptoms of generalized tetanus. The horse died in twenty hours.

*Autopsy.* No skin lesions; mucous membrane of the right sac of the stomach and cæcum inflamed; small ulceration at pyloric end of duodenum; mucous membrane of colon and rectum red and ecchymotic, and in some places red points as if made with a pin (parasitic lesion); two ulcerations, 3 to 4 mm. in diameter; twenty oxyuris, still living, some attached and some free in the intestinal contents of the diaphragmatic curvature of the large colon; lungs congested.

A portion of the hay distributed among the regiment came from a region where tetanus had been almost epizoötic.

The author believes that the lesions of the intestine gave passage to the toxin of the bacillus of Nicolier, and strengthens his opinion by recalling two previous cases of this kind that tetanus, like other microbic maladies, may be communicated through the digestive tract.—*Rec. de Med. Vet.*

TREATMENT OF RHEUMATISM. Frinchera recommends, besides the usual internal remedies, the subcutaneous injection over the shoulder of a saturated solution of chlorate of potassium. The skin is shaved and disinfected; at from 12 to 20 points, 2 to 2½ drachms are injected. This produces considerable inflammation, which disappears in from three to four weeks.—*Thierärztliches Centralblatt.*

